# Prevalence and correlates of eating disorders in bipolar patients

**DOI:** 10.1192/j.eurpsy.2025.1383

**Published:** 2025-08-26

**Authors:** M. Abdelkefi, S. Ellouze, W. Abid, R. Jbir, N. Bouattour, N. Halouani, M. Turki, J. Aloulou

**Affiliations:** 1 Psychiatry B, Hedi Chaker university hospital, Sfax, Tunisia

## Abstract

**Introduction:**

Bipolar disorder is a chronic mood disorder that often coexists with a range of psychiatric and physical comorbidities. Among these, eating disorders (ED) have emerged as a significant concern due to their impact on the course and prognosis of BD.

**Objectives:**

This study aims to investigate the prevalence of ED in stabilized bipolar patients and identify clinical and demographic factors associated with this comorbidity.

**Methods:**

We conducted a cross-sectional, descriptive, and analytical study of patients followed for bipolar disorder at the psychiatry outpatient unit at the Hedi Chaker University Hospital in Sfax. The questionnaire included sociodemographic data, medical and psychiatric history, and anthropometric characteristics. Eating disorders were assessed using the Eating Attitude Test 40 (EAT-40).

**Results:**

Our study included 93 patients. The mean age was 41.49±12.33 years, with a M/F sex ratio of 2.58. Among the patients, 58.1% were married, 45.2% had secondary education, and 47.3% were unemployed. Personal somatic history was reported by 35.5% of participants, and 11.8% had psychiatric comorbidities alongside bipolar disorder.

The mean body mass index (BMI) was 27.4 kg/m² (SD=5.96). Of the patients, 29% were overweight, and 31.2% were obese. The prevalence of eating disorders was 18.3%.

EAT-40 scores were significantly associated with age (p=0.018), female gender (p<10⁻³), living alone (p=0.031), lack of formal education (p=0.009), history of medical comorbidities (p<10⁻³), weight (p=0.05), and BMI (p=0.003).

**Conclusions:**

The significant associations between EAT-40 scores and sociodemographic factors underscore the need for targeted screening and interventions in this population. Early detection and management of eating disorders in bipolar patients are crucial to improving clinical outcomes and overall quality of life.

**Disclosure of Interest:**

None Declared

